# Hepatic IGF2/H19 Epigenetic Alteration Induced Glucose Intolerance in Gestational Diabetes Mellitus Offspring *via* FoxO1 Mediation

**DOI:** 10.3389/fendo.2022.844707

**Published:** 2022-04-01

**Authors:** Ying Jiang, Hong Zhu, Zi Chen, Yi-Chen Yu, Xiao-Han Guo, Yuan Chen, Meng-Meng Yang, Bang-Wu Chen, Matthew Sagnelli, Dong Xu, Bai-Hui Zhao, Qiong Luo

**Affiliations:** ^1^ Department of Obstetrics, Women’s Hospital, Zhejiang University, School of Medicine, Hangzhou, China; ^2^ Obstetrics and Gynecology Hospital, Institute of Reproduction and Development, Fudan University, Shanghai, China; ^3^ Department of General Surgery, Sir Run Run Shaw Hospital, Zhejiang University, School of Medicine, Hangzhou, China; ^4^ Department of Obstetrics, Ninghai Maternal and Child Health Hospital, Ningbo, China; ^5^ University of Connecticut School of Medicine, Farmington, CT, United States

**Keywords:** gestational diabetes mellitus (GDM), epigenetic regulation, DNA methylation, fetal-origin diseases, insulin resistance

## Abstract

**Objective:**

The offspring of women with gestational diabetes mellitus (GDM) have a high predisposition to developing type 2 diabetes during childhood and adulthood. The aim of the study was to evaluate how GDM exposure in the second half of pregnancy contributes to hepatic glucose intolerance through a mouse model.

**Methods:**

By creating a GDM mouse model, we tested glucose and insulin tolerance of offspring by intraperitoneal glucose tolerance test (IPGTT), insulin tolerance test (ITT), and pyruvate tolerance test (PTT). In addition, we checked the expression of genes IGF2/H19, FoxO1, and DNMTs in the mouse liver by RT-qPCR. Pyrosequencing was used to detect the methylation status on IGF2/H19 differentially methylated regions (DMRs). *In vitro* insulin stimulation experiments were performed to evaluate the effect of different insulin concentrations on HepG2 cells. Moreover, we detect the interaction between FoxO1 and DNMT3A by chromatin immunoprecipitation–quantitative PCR (Chip-qPCR) and knock-down experiments on HepG2 cells.

**Results:**

We found that the first generation of GDM offspring (GDM-F1) exhibited impaired glucose tolerance (IGT) and insulin resistance, with males being disproportionately affected. In addition, the expression of imprinted genes IGF2 and H19 was downregulated in the livers of male mice *via* hypermethylation of IGF2-DMR0 and IGF2-DMR1. Furthermore, increased expression of transcriptional factor FoxO1 was confirmed to regulate DNMT3A expression, which contributed to abnormal methylation of IGF2/H19 DMRs. Notably, different insulin treatments on HepG2 demonstrated those genetic alterations, suggesting that they might be induced by intrauterine hyperinsulinemia.

**Conclusion:**

Our results demonstrated that the intrauterine hyperinsulinemia environment has increased hepatic FoxO1 levels and subsequently increased expression of DNMT3A and epigenetic alterations on IGF2/H19 DMRs. These findings provide potential molecular mechanisms responsible for glucose intolerance and insulin resistance in the first male generation of GDM mice.

## Introduction

Gestational diabetes mellitus (GDM) is defined as glucose intolerance first detected during pregnancy and is one of the most common complications of pregnancy (1). The estimated prevalence of GDM is about 15% among pregnant women (2). Increasing evidence indicates that maternal GDM influences their offspring’s health, notably resulting in a higher risk of cardiometabolic diseases such as type 2 diabetes and obesity (3, 4). Currently, there are no definitive treatments for GDM except lifestyle change and limited insulin therapy (5). Therefore, we aimed to explore the potential mechanisms related to the impact of GDM on the long-term health of the offspring.

Recently, an increasing field of data articulates an interest in the potential mechanism between epigenetic regulation and the susceptibility to chronic cardiometabolic diseases in GDM offspring (6, 7). DNA methylation is the most extensively studied epigenetic modification. Hjort et al. (8) performed a genome-wide DNA methylation study on peripheral blood in adolescent offspring (9–16 years old) of GDM women, and the results indicated that intrauterine exposure to hyperglycemia was related to DNA abnormal methylation in 76 differentially methylated CpG sites (8). Cote et al. (9) put forward that DNA hypermethylation of peroxisome proliferator-activator receptor-gamma, co-activator 1, alpha (PPARGC1α) in the placenta was involved in the association between maternal hyperglycemia and elevated cord blood leptin levels, which is considered as a marker for adiposity in offspring (9). Ding et al. (10) found that the expression of imprinted gene IGF2/H19 was downregulated in mouse pancreatic islets of GDM offspring due to hypermethylation status in the differentially methylated region (DMR) (10).

Normal pregnancy is typically a state of insulin resistance because of a surge of placental anti-insulin hormones (11). Moreover, hyperglycemia in GDM patients is induced by insulin secretion that is inadequate to compensate for the concurrent insulin resistance. Therefore, chronic hyperinsulinemia is a crucial element of the pathophysiology of GDM (12). However, the concrete mechanism between intrauterine exposure to hyperinsulinemia and diabetes offspring is limited, since most studies have focused on hyperglycemia in the intrauterine environment (13, 14).

Under normal physiological feeding conditions, insulin canonically modulates hepatic gluconeogenesis to lower glucose concentration by combining its receptor (IR) and insulin receptor substrates (Irs) and then initiating the phosphorylation of Akt protein kinases (15, 16). Forkhead box O1 (FoxO1) inhibits the canonical Akt-induced insulin pathway as a critical liver transcription factor in reducing genes coding gluconeogenic enzymes (17, 18). Accordingly, FoxO1 is active during fasting by collaborating with co-activators PPARGC1α to coordinatively upregulate the expression of G6pc (glucose-6-phosphatase, catalytic subunit) and Pck1 (cytosolic phosphoenolpyruvate carboxykinase 1) (19, 20) and is suppressed by Akt-mediated phosphorylation after feeding (21). FoxO1 knockout in the mouse livers could afford an improvement in glucose homeostasis and subsequently reduce insulin resistance (16).

Consequently, in our study, we hypothesized that intrauterine hyperinsulinemia in GDM induced abnormal DNA methylation through insulin pathway transcription factor FoxO1, which subsequently causes abnormal expression of imprinted gene IGF2/H19, ultimately resulting in glucose intolerance and insulin resistance in adult GDM offspring.

## Materials and Methods

### Animal Care and Model

**Figure 1:**
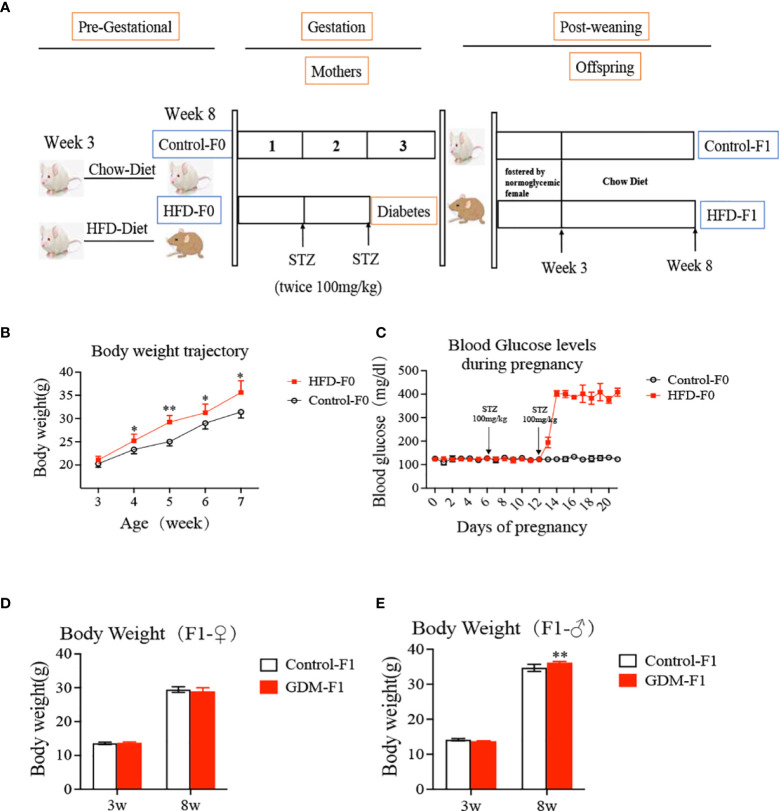
Experimental design, offspring growth curves. **(A)** Experimental design; the ICR female mice (age 3-4 weeks) divided into two group, fed with a high-fat diet or chow diet for 4 weeks. Then the mice were mated with nonnal ICR males. The pregnancy ICR females were injected STZ or citrate buffer on day 6 and day 12 of pregnancy. Both the control and GDM pups were fostered by normoglycemic female until they were three weeks old, and then fed by chow diet. **(B)** body weight of mice both fed by high fat diet or chow diet at different developmental weeks; **(C)** Blood glucose levels during pregnancy for mice both fed by high fat diet or chow diet; **(D)** Body weight of female F1 offspring; **(E)** Body weight of male F1 offspring. In all panels, data are presented as mean ± SD, *P < 0.05. **P < 0.01, n = 6 mice per group. Significance was determined by Student *t* test.

The Zhejiang University Animal Care and Use Committee (IACUC) approved all the animal care and treatment protocols (ZJU2015-323-01). All the experiments were conducted by the Institute of Cancer Research (ICR) mice, which were purchased from Shanghai SLAC Laboratory Animal Company (Shanghai, China). Virgin and healthy ICR females (age 3–4 weeks) were fed with a high-fat diet (HFD) containing 60 kcal% fat (D12492, Research Diets, New Brunswick, NJ, USA) or chow diet. The mice were then mated with normal ICR males. A copulation plug present overnight was considered a pregnancy (day 0.5). The pregnant ICR females were divided into two groups, control (Ctrl) and GDM, and fed with an HFD during the whole course of pregnancy. On day 6 and day 12 of pregnancy, GDM dams were fasted 8 h and received a streptozotocin (STZ) injection (100 mg/kg i.p.) (Sigma-Aldrich, St. Louis, MO, USA) (22, 23) (**Figure 1A**). Control dams received an equal volume of citrate buffer. We tested the blood glucose level *via* the tail vein 48–72 h after the second STZ injection, and diabetes was defined as a blood glucose level over 14 mmol/L (252 mg/dl) (24), which is confirmed by continuous monitoring in the following days. Both the control and GDM pups were fostered by normoglycemic females until they were 3 weeks old. Mice were weighed at ages 3, 8, and 16 weeks.

### Glucose, Insulin, and Pyruvate Tolerance Tests

Glucose tolerance tests (GTTs) were performed in overnight (16 h) fasted animals as previously described (25). Each mouse was administered an intraperitoneal (i.p.) injection of 2 g/kg body weight (BW) glucose (DeltaSelect, Munich, Germany) in sterile saline. Blood glucose concentrations were measured collection from the tail vein at 30, 60, and 120 min after the injection.

Insulin tolerance tests (ITTs) were performed in *ad libitum* fed mice as the previous study reported (26919426). Each animal received an i.p. injection of insulin at 0.8 U/kg (Humulin R; Eli Lilly, Indianapolis, IN, USA). Blood glucose levels were then measured at 30, 60, and 120 min after insulin injection.

Pyruvate tolerance test (PTT) was performed after overnight fasting of 12 h. Animals received an i.p. injection of 1.5 g/kg BW sodium pyruvate (Cat#P5280, Sigma-Aldrich, St. Louis, MO, USA). Blood glucose levels were then measured at 30, 60, and 120 min after the injection. The area under the curve (AUC) of glucose against time was calculated in order to analyze glucose tolerance as previous studies described (10, 26).

### Serum Analysis

Serum was collected from 16-week-old mice by fasting for 12 h. Serum insulin level was evaluated at an overnight state (Crystal Chem, Downers Grove, IL, USA). HOMA-insulin resistance (IR) was calculated as our previous study reported (22): fasting serum insulin concentration (μU/ml) multiplied by fasting blood glucose level (mg/dl) divided by 405.

### RNA Extraction and Quantitative PCR Analysis

Total RNA was extracted from the mouse liver tissue samples (16 weeks old) and fetal liver tissue (day 18.5) with the TRIzol Reagents (Invitrogen Life Technologies, Carlsbad, CA, USA). Complementary DNA was synthesized using oligo-deoxythymidylic acid and random primers (RR037A, Takara, Maebashi, Japan). Quantitative PCR was performed using ABI Prism 7900HT (Applied Biosystems, Foster City, CA, USA) with SYBR green detection (RR420A, Takara, Japan). Glyceraldehyde-3-phosphate dehydrogenase was the internal control. The primer sequences are provided in **Supplementary Table 1**.

### DNA Isolation and Bisulfite Conversion

Total genomic DNA was isolated from the mouse liver tissue by using the Genomic DNA Purification Kit (Cat. K0512, Invitrogen Life Technologies). Bisulfite conversion was performed by the EpiTect bisulfite kit (Qiagen, Valencia, CA, USA) according to the manufacturer’s instructions; the details are shown in a previous study (27).

### DNA Methylation Analysis by Pyrosequencing

The methylation status was analyzed by pyrosequencing (27). In brief, pyrosequencing primers were designed by Qiagen PyroMark Assay Design 2.0 software (Qiagen) (**Supplementary Table 2**). PCR product was checked by agarose gel analysis. Pyrosequencing was carried out on a PyroMark Q96 instrument (Qiagen) according to the manufacturer’s instruction. The Pyro Q CpG software (Qiagen) was used to calculate the percentage of methylation.

### Cell Culture, Insulin Treatment, and siRNA Transfection

The established human HepG2 hepatoma cell line was obtained from the American Type Culture Collection (Rockville, MD, USA). The HepG2 cells were cultured in Dulbecco’s modified Eagle’s medium (DMEM; Gibco, Beijing, China), supplemented with 10% fetal bovine serum (FBS) (Gibco, Beijing, China) and 1% penicillin and streptomycin (PS) at 37°C in 5% humidified CO_2_ tissue culture incubator. Generally, the cells were seeded in 24-well plates for 12 h and then incubated in an FBS-free DMEM for 24 h, followed by different insulin concentrations (0, 50, 100 nmol/L) treatment for 24h.

For siRNA transfection, the sequences of siRNA targeting FoxO1 (siFoxO1) are as follows (5′–3′): sense: GCAGCAGACACCAUGCUAUTT; anti-sense: AUAGCAUGGUGUCUGCUGCTT. The sequences of negative control (siCon) are as follows (5′–3′): UUCUCCGAACGUGUCACGUdTdT; anti-sense, ACGUGACACGUUCGGAGAAdTdT. siFoxO1 or siCon was mixed with 25 μl of OPTI-MEM (Cat. 31985-070, Gibco) by gentle pipetting. Meanwhile, 1 μl of Lipofectamine 3000 reagent (Cat. L3000008, Thermo Fisher Scientific, Waltham, MA, USA) was mixed with 25 μl of OPTI-MEM. Then the Lipofectamine or siRNA from two tubes was mixed together and incubated for 5 min at room temperature (RT). Then cell pellet (5 * 10^4^ cells) was resuspended in a total 50 μl solution and incubated at RT for 10 min, 450 μl of DMEM containing FBS was added, and the cell suspension was transfected into one well of a 24-well plate.

### Western Blotting

The protein was extracted from HepG2 cells with lysis buffer, which was separated using 10% sodium dodecyl sulfate–polyacrylamide gel electrophoresis (SDS-PAGE). Western blotting was performed using polyvinylidene fluoride membrane and the antibodies for FoxO1 (Cell Signaling Technology, Danvers, MA, USA; 2880, used at a dilution 1:1,000), DNMT3A (Cell Signaling, 32578, used at a dilution of 1:1,000), and Actin (Abcam, Cambridge, UK; ab8227, used at a dilution 1:1,000). The enhanced chemiluminescence system (Pierce, Rockford, IL, USA) was used to visualize protein bands.

### Chromatin Immunoprecipitation–Quantitative PCR

The detailed protocol is described in a previous study (28). Briefly, all experiments were performed on a 10-cm plate scale by Chromatin Prep Module Kit (Cat. 26158, Thermo Fisher Scientific) according to the manufacturer’s instructions. Agarose beads were used to pre-bind overnight with antibodies against FoxO1 (Abcam, ab39670, used at a dilution of 1:500). HepG2 cells were cross-linked with 1% formaldehyde at RT for 10 min and then stopped by 1* glycine. Chips were performed at 4°C. Primers for the specific promoter regions of DNMT3A are provided in **Supplementary Table 3**. The relative enrichment of the indicated DNA regions was measured according to the manufacturer’s instruction and was normalized to % input.

### Statistical Analysis

All data were presented as mean ± SD. Statistical analysis was performed by two-tailed Student’s *t*-test and one-way ANOVA as described in the table and figure legends, using SPSS 18.0 software. p < 0.05 and p < 0.01 were considered statistically significant.

## Results

### Gestational Diabetes Mellitus Environment Induced Increased Birth Weight in Male Adult Offspring

We established a mouse model of hyperglycemia during the midlate stage of pregnancy mimicking the clinical GDM. The bodyweight of HFD-fed mice was significantly higher than that of chow diet-fed mice (**Figure 1B**), while plasma glucose concentrations at day 0 were similar in two groups (**Figure 1C**). After two lose-dose STZ injections, the plasma glucose levels of GDM dams averagely reached 403 mg/dl (**Figure 1C**). Notably, increased BW was seen in GDM-F1 male adult offspring at 8 weeks of age (**Figure 1E**). However, these associations were not observed in GDM-F1 female adult offspring (**Figure 1D**).

### Gestational Diabetes Mellitus Exposure Induced Glucose Intolerance, Insulin Resistance, and Enhanced Gluconeogenesis in GDM-F1 Offspring

**Figure 2:**
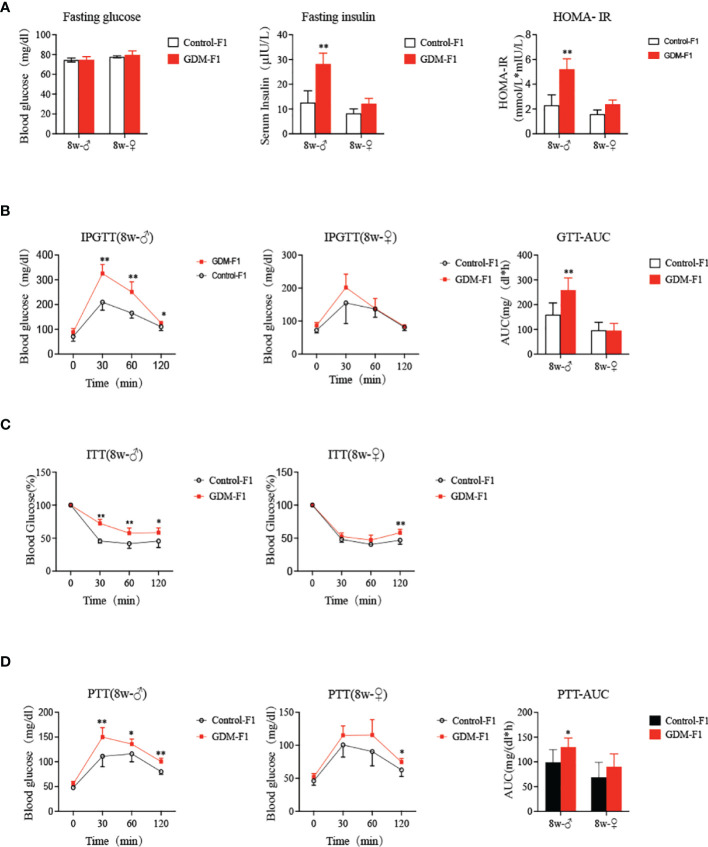
Glucose Homeostasis on GDM-F1 and control-F1 mice. **(A)** fasting serum glucose concentration, fasting insulin concentration, HOMA-IR of 8-week-old F1 offspring; **(B)** Glucose tolerance test (GTT, 2 g/kg glucose ip) and Area under the curve (AUC) of 8-week-old F1 offspring over the course of 120 min; **(C)** insulin tolerance test (ITT, 0.75 U/kg insulin ip) of 8-week-old F1 offspring over the course of 120 min; **(D)** Pyruvate tolerance test (PTT, 2g/kg pyruvate ip) and AUC of 8-week-old F1 offspring over the course of 120 min; data are presented as mean ± SD, *P < 0.05. **P < 0.01, n = 6 mice per group. Significance was determined by Student *t* test.

Fasting blood glucose levels of 8-week-old GDM-F1 offspring did not differ from those in the corresponding controls, and there was no gender difference (**Figure 2A**). We further performed a GTT by i.p. injection of glucose (2 g/kg body wt). There was no difference in the glucose levels and GTT AUC in 8-week-old female offspring between GDM-F1 and Control-F1 (**Figure 2B**). However, impaired glucose tolerance (IGT) was found in both 8-week-old mice of the GDM-F1 group, whose blood glucose level significantly increased at 30 min after injection (**Figure 2B**); along with this, we found increased fasting insulin concentration in GDM-F1 male mice (**Figure 2A**). In addition, decreased insulin sensitivity was observed in 8-week-old male mice of the GDM-F1 group in the ITT. Interestingly, the difference between GDM-F1 and Control-F1 female mice was not obvious (**Figure 2C**), consistent with the results of HOMA-IR (**Figure 2A**). Regarding hepatic gluconeogenic activity, the PTT displayed that the GDM-F1 male mice showed enhanced blood glucose levels compared with the Control-F1 group (**Figure 2D**). In contrast, excessive gluconeogenesis was not observed in 8-week-old female GDM-F1 mice (**Figure 2D**).

### Intrauterine Hyperinsulinemia Downregulated the Expression of IGF2 and H19 in Male Mouse Liver

**Figure 3:**
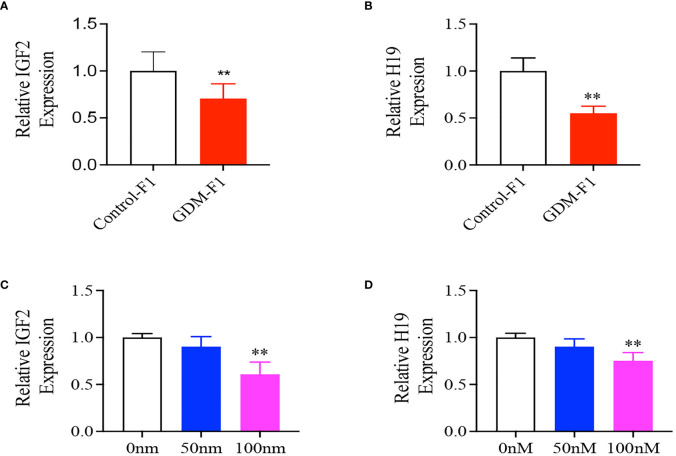
Representative mRNA levels of Igf2, H19 in the liver of 8-week-old F1 male mice and HepG2 cells treated with different insulin concentration. **(A, B)** IGF2/H19 mRNA expression in the liver of 8-week-old mice (every group contains 10 mice); **(C, D)** IGF2, H19 gene expression in HepG2 cells treated with 0, 50, 100 mnol/L insulin concentration (n = 4 replicates/group in at least three independent isolations). Data were analyzed with the Eq. 2^-ΔΔCT^ where ΔΔCT = ΔCT (treatment group) -ΔCT (control group), and ΔCT = ΔCT (sample) - ΔCT (intemal control). The values were normalized to ACTIN mRNA levels, ACTIN was an intemal control. Data are presented as mean ± SD, *P < 0.01. Significance was determined by Student *t* test.

We collected the liver from 8-week-old male mice and found that the relative mRNA levels of IGF2 and H19 were both significantly lower in GDM-F1 male mice (**Figures 3A, B**), which was consistent with the islet results previously published in *Diabetes* (10). In order to verify the direct effect of hyperinsulinemia on liver development, HepG2 cells were cultured in a medium containing different insulin concentrations for 24 h. We found that the IGF2 and H19 mRNA levels were significantly lower in HepG2 cells exposed to a high insulin concentration (100 mmol/L) than those in the control or a low concentration of insulin (50 nmol/L) (**Figures 3C, D**).

### Intrauterine Hyperinsulinemia Induced Hypermethylation of IGF2/H19 Differentially Methylated Region in Male Mouse Liver

**Figure 4:**
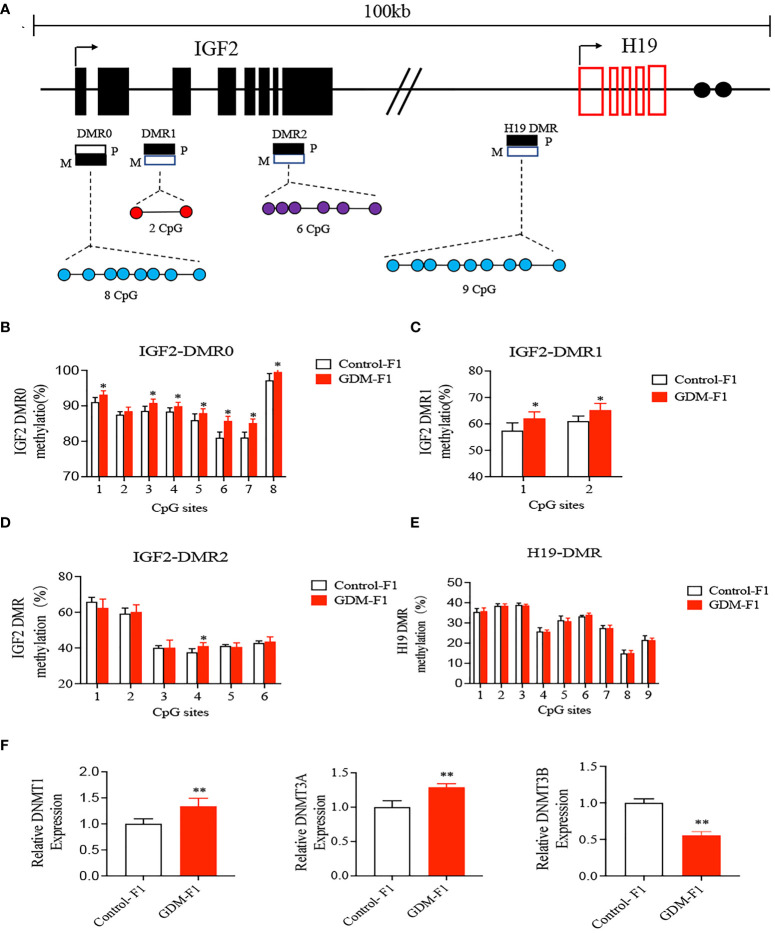
Methylation analysis of IGF2/H19 DMRs by pyrosequencing. **(A)** Schematic representation of mouse imprinted locus, showing the relative positions of the IGF2 and H19 genes and indicating the location of the four DMR knows to contribute to IGF2 imprinting. Enhancers **(E)** are indicated as black circles. The locations of the four DMRs within the IGF2/H19 imprinted locus represented by boxes are shaded to indicate preferential methylation of the maternal (M) or paternal (P) allel in each region. **(B–E)** Percentage of methylation for each CpG cytosine is calculated. **(B)** Methylation status of individual DNA strands of IGF2-DMR0 containing 8 CpG sites; **(C)** IGF2-DMRI containing 2 CpG sites; **(D)** IGF2-DMR2 containing 6 CpG sites; **(E)** H19-DMR containing 9 CpG and the average methylation ratio in each CpG site. **(F)** The relative gene expression of DNMTI, DNMT3A, DNMT3B in the liver of 8-week-old mice. Data were analyzed with the Eq. 2^-ΔΔCT^, where ΔΔCT = ΔCT (treatment group) -ΔCT (control group), and ΔCT = ΔCT (sample) -ΔCT (internal control). The values were normalized to ACTIN mRNA levels. For pyrosequencing and RT-qPCR, every group contains 10 mice. In all panels, data are presented as mean ± SD, *P < 0.05, **P < 0.01, Significance was determined by Student *t* test.

As imprinted genes, IGF2 and H19 allelic expressions in mice are regulated by allele-specific methylation at four DMRs (10, 29) (**Figure 4A**). We collected liver from a 16-week-old male of the control and GDM-F1 groups. We analyzed the methylation levels of 8 cytosine phosphate guanine (CpGs) of the IGF2-DMR0, 2 CpGs of the IGF2-DMR1, 6 CpGs of the IGF2-DMR2, and 9 CpGs of the H19-DMR by pyrosequencing. In the IGF2-DMR0 and IGF2 DMR1, the methylation status was significantly higher in GDM-F1 groups compared with that in control except for site 2 CpG in IGF2-DMR0 (**Figures 4B, C**). Furthermore, only site 4 CpG exhibited significant hypermethylation in IGF2-DMR2 of GDM-F1 liver (**Figure 4D**). Though the H19 DMR upstream of the H19 gene (located 90 kb 3′ of IGF2) acted as a methylation-sensitive boundary component, there was no significant difference in these two groups (**Figure 4E**).

DNA methylation is processed by a family of enzymes called the DNA methyltransferases (DNMTs), which include DNMT1, DNMT3A, and DNMT3B (30). DNMT1 is conservatively expressed and responsible for the maintenance of methylation, while DNMT3A and DNMT3B are required for *de novo* methylation (31). We performed qPCR to detect the expression of DNMTs, and the results indicated that there was a significant elevation of DNMT1 and DNMT3A, while there was a significant reduction of DNMT3B in GDM-F1 mouse livers compared with Control-F1 mouse livers (**Figure 4F**).

### Intrauterine Hyperinsulinemia Induced Elevated Expression of DNMT3A *via* Activating FoxO1 Expression

**Figure 5:**
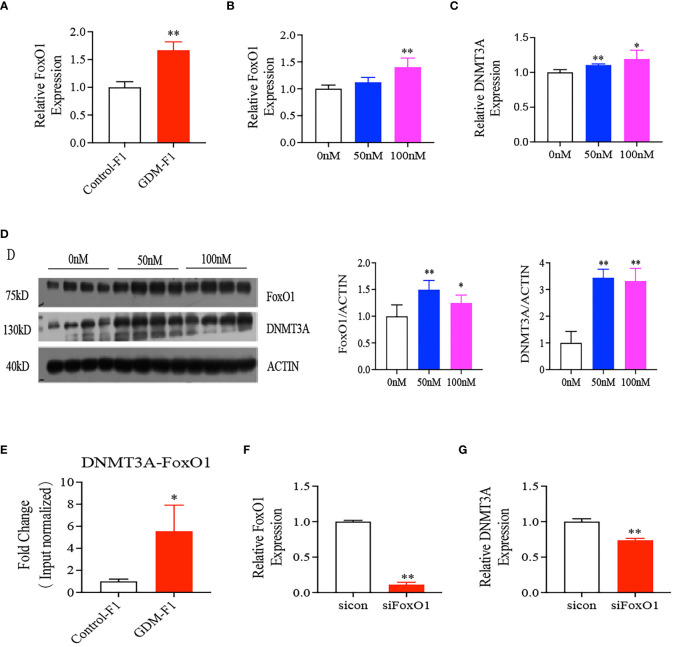
Interaction between FoxO1 and DNMT3A. **(A)** Representative mRNA levels of FoxO1 in the liver of 8-week-old mice (every group contains 10 mice); **(B–D)** FoxO1, DNMT3A gene expression in HepG2 cells treated with 0, 50, 100 nmol/L insulin concentration after 24 hours by RT-qPCR or Western Blot (n = 4 replicates/group in at least three independent isolations); **(E)** chip-qPCR analysis to detect binding between FoxO1 and DNMT3A. IgG was as negative control. **(F, G)** mRNA level of FoxO1 and DNMT3A after silence interfere FoxO1 on HepG2 cells (n = 4 replicates/group in at least three independent isolations); For RT-qPCR, Data were analyzed with the Eq. 2-ΔΔCT, where ΔΔCT = ΔCT (treatment group) - ΔCT (control group), and ΔCT = ΔCT (sample) - ΔCT (internal control). The values were normalized to ACTIN mRNA levels, ACTIN was an internal control. In all panels, data are presented as mean ± SD, *P < 0.05, **P < 0.01. Significance was determined by Student *t* test.

FoxO1 plays a major role in the regulation of insulin sensitivity, and the liver is one of the critical sites of action. In our study, we found that the relative expression of FoxO1 significantly increased in the GDM-F1 male mice through RT-qPCR compared to Control-F1 male mice (**Figure 5A**). Chromatin immunoprecipitation coupled with qPCR (Chip-qPCR) demonstrated the association between FoxO1 with DNMT promotor region. The binding of FoxO1 onto the promotor of DNMT3A was increased in the 8-week-old liver of GDM-F1 male mice compared to that in Control-F1 male mice (**Figure 5E**). In order to evaluate whether intrauterine hyperinsulinemia induced the abnormal expression of FoxO1 and DNMT3A, we cultured HepG2 in different insulin concentrations, and the results indicated that the expression of FoxO1 and DNMT3A gradually augmented as the insulin concentration increased, at both the mRNA and protein levels (**Figures 5B–D**). In addition, we also measured the effect of FoxO1 deletion on DNMT3A in the HepG2 cells. The DNMT3A level was significantly decreased in HepG2 cells following injection of si-FoxO1 (**Figures 5F, G**). These results indicated that elevated expression of FoxO1 increased the DNMT3A level through binding to its promoter region, which might be responsible for hypermethylation of the IGF2/H19 DMR region.

## Discussion

In this study, we demonstrated that the male offspring exposed to GDM environment developed significantly exacerbated glucose intolerance and insulin resistance as compared with the females. Similar observations have been made in a previous study (22). Downregulated IGF2/H19 gene expression in the liver of GDM male offspring can be explained by abnormal DNA methylation due to increased expression of DNMT3A. The IGF2/H19 epigenetic signature was already present in sperm samples from the paternal line (10). In turn, transcription factor FoxO1 binding in the promoter of DNMT3A resulted in altered DNMT3A expression. *In vitro* insulin stimulation experiment also verified the interaction between DNMT3A and FoxO1. Hence, our data strongly addressed the effect of hyperinsulinemia environment on GDM offspring.

The Diabetes in Pregnancy Study in Chicago revealed that offspring of diabetic mothers (age ranging from 10 to 16 years) had a significantly higher prevalence of IGT than the sex- and age-matched control groups (32). Boerschmann et al. (33) recruited 232 offspring of mothers with GDM (OGDM) and 431 offspring of non-diabetic mothers (ONDM), and they found the HOMA-IR was significantly increased in the OGDM group compared with those with ONDM mothers (33). This evidence demonstrates that maternal hyperglycemia in GDM confers a major risk of diabetes in exposed offspring (34). In addition, a number of diabetes-related animal models confirmed the phenomenon observed in clinical practice (10, 22). In our study, although there was no difference in the extent of fasting glucose in the offspring of GDM, blood glucose levels significantly increased at 30 or 60 min after injection in GDM offspring, especially for male mice, which indicated that GDM male mice demonstrated glucose intolerance in their adulthood. Moreover, after insulin injection, glucose levels in the male offspring exposed to GDM exhibited the tendency of slow declination and fast rising. This evidence strongly shows that male mice exposed to GDM have a manifestation of insulin resistance, and those glucose disturbances have a marked sex difference, in accordance with a previous study (10).

The liver is a critical organ during insulin-mediated modulation of metabolism, in particular glucose and lipid homeostasis. Insulin-like growth factor II (IGF2) is confirmed to be involved in stimulating glycogen synthesis in fetal hepatocytes through the IGF2-deficient mouse model (35). Furthermore, injecting IGF2 into hypophysectomized rats would induce decreased blood glucose and increased hepatic glycogen synthesis (36, 37). H19 locates downstream 90 kb of IGF2 on mouse chromosome 7 and is reciprocally imprinted. Downregulation of H19 stimulates an increase in the expression of gluconeogenic genes *via* FoxO1, subsequently resulting in increased glucose output (38, 39). In addition, decreased levels of H19 have been documented in insulin-resistant mice, which inhibit the expression of key metabolic genes by increasing the bioavailability of let-7 (40). Considering the important regulation of IGF2/H19 on the hepatic glucose metabolism, we detected the expression of IGF2/H19 on mouse livers. We found downregulation of IGF2/H19 expression in GDM-F1 mice compared with control mice, together with increased gluconeogenesis by PTT. Meanwhile, *in vitro* culture experiments confirmed the effect of insulin stimulation on IGF2/H19 in HepG2 cells, providing a potential explanation for intrauterine hyperinsulinemia directly affecting IGF2/H19 and subsequent impaired glucose metabolism.

Dabelea et al. (3) enrolled nuclear families in which at least one sibling was born before and one after the mother was diagnosed with type 2 diabetes and found that the sibling born after their mother displayed diabetes had a 3.7-fold higher risk of diabetes (3). The results emphasized the effects of abnormal *in utero* environments on fetuses beyond direct genetic transmission. DNA methylation without involving alteration of DNA sequences is susceptible to environmental stimuli such as toxic substances and thereby plays a critical role in elaborating the potential mechanisms of impaired glucose metabolism (41). The mouse IGF2 and H19 genes were regulated by allele-specific methylation at four DMRs: IGF2-DMR0, IGF2-DMR1, IGF2-DMR, and H19-DMR (42). Since glucose disturbance appeared much more prominent in male offspring, we examined all of the IGF2/H19 DMRs in the liver of GDM-F1 male mice. Our study indicated that *in utero* exposure to GDM could induce hypermethylation at IGF2-DMR0 and IGF2-DMR1 in GDM-F1 male offspring.

The transcriptional factor FoxO1 is an important regulator of key gluconeogenesis enzymes in the liver (43). In the fed state, insulin signaling stimulates Akt phosphorylation, subsequently activating FoxO1 expression, consequently increasing transcriptional induction of two gluconeogenic enzymes, glucose-6-phosphatase catalytic subunit (G6Pc) and phosphoenolpyruvate carboxykinase (PEPCK) (15, 44). Therefore, we detected the FoxO1 expression in our diabetes mouse model and found that GDM-F1 male mice showed increased expression of FoxO1, and *in vitro* experiments confirmed that exogenous insulin stimulation produced a similar tendency of FoxO1 as those *in vivo*. In addition, FoxO1 mediates targeted genes by binding to the FoxO-binding element as a transcriptional factor, such as uncoupling protein 1 (Ucp1) (45), peroxisome proliferator-activated receptor-alpha (PPARα) (46). However, by far, few studies have focused on the relationship between FoxO1 and DNMTs, which is a prerequisite for DNA methylation. In our study, through Chip-qPCR and *in vitro* knock-down experiments, we found that FoxO1 might regulate the expression of DNMT3A, subsequently inducing epigenetic alterations in the GDM-F1 male mice.

In conclusion, our study demonstrated that intrauterine hyperinsulinemia increased hepatic FoxO1 levels and contributed to the upregulation of DNMT3A, subsequently inducing the downregulation of IGF2/H19 expression in the liver of GDM-F1 mice. These findings provide insights into the molecular mechanisms responsible for glucose intolerance and insulin resistance in first-generation males of GDM mice.

## Data Availability Statement

The original contributions presented in the study are included in the article/**Supplementary Material**. Further inquiries can be directed to the corresponding author.

## Ethics Statement

The animal study was reviewed and approved by the Zhejiang University Animal Care and Use Committee (IACUC).

## Author Contributions

YJ contributed to the collection, analysis, and interpretation of data as well as manuscript preparation. HZ contributed to the animal model establishment. ZC contributed to the molecular experiments. X-HG, Y-CY, and YC contributed to the data collection and analysis. M-MY, B-WC, and DX contributed to the interpretation of data. MS contributed to the language editing. B-HZ and QL contributed to the study design, data interpretation, and manuscript preparation. QL is the guarantor of this work and, as such, has full access to all the data in the study and takes responsibility for the integrity of the data and the accuracy of the data analysis. All authors listed have made a substantial, direct, and intellectual contribution to the work and approved it for publication.

## Funding

This work was supported by the Natural Science Foundation of Zhejiang Province (LQ20H040008, LY20H040009), Scientific Research Foundation of the National Health Commission (WKJ-ZJ-2126), National Nature Science Foundation of China grant 81571447, and Key Project of Science and Technology Department of Zhejiang University Province (2018C03010). The National Nature Science Foundation of China (Grant No. 82001645) HZ received these funding.

## Conflict of Interest

The authors declare that the research was conducted in the absence of any commercial or financial relationships that could be construed as a potential conflict of interest.

## Publisher’s Note

All claims expressed in this article are solely those of the authors and do not necessarily represent those of their affiliated organizations, or those of the publisher, the editors and the reviewers. Any product that may be evaluated in this article, or claim that may be made by its manufacturer, is not guaranteed or endorsed by the publisher.
