# Supplementary material for: Hepatic IGF2/H19 Epigenetic Alteration Induced Glucose Intolerance in Gestational Diabetes Mellitus Offspring via FoxO1 Mediation
Source: Front Endocrinol (Lausanne). 2022 Apr 1;13:844707. doi: 10.3389/fendo.2022.844707 (PMC9011096; doi:10.3389/fendo.2022.844707)
Supplement: Supplementary file 1 [file DataSheet_1.docx]

**Supplementary Table 1. Nucleotide sequences of primers (mouse) used for real-time quantitative PCR (SYBR Green)**

| Target RNA | Primers (5’ to 3’ direction) | Product Size |
| --- | --- | --- |
| FoxO1 | F: TTTCGTCCTCGAACCAGCTC | 107bp |
|  | R: TACACCAGGGAATGCACGTC |  |
| IGF2 | F: TGCCCATTTGGCTGGACA | 136bp |
|  | R: AGGTGCCTGCATCAAGGTGAC |  |
| H19 | F: GCACTAAGTCGATTGCACTGG | 164bp |
|  | R: GCCTCAAGCACACGGCCACA |  |
| β-actin | F: AGTGTGACGTTGACATCCGT | 423bp |
|  | R: GCAGCTCAGTAACAGTCCGC |  |
| DNMT1 | F: GTCTTCCCCCACTCTCTTGC | 134bp |
|  | R: ATCTTGCAGGTTGCAGACGA |  |
| DNMT3A | F: GGGAGGGCACAGCAAGG | 189bp |
|  | R: TGCCGTGGTCTTTGTAAGCA |  |
| DNMT3B | F: GCCAGACCTTGGAAACCTCA | 150bp |
|  | R: GCTGGCACCCTCTTCTTCAT |  |

**Supplementary Table 2 Nucleotide sequences of primers (mouse) used for pyrosequencing PCR**

| Methylation Analysis | | Primers |
| --- | --- | --- |
| IGF2-  DMR0 | Primer1 | F: [Btn]TTTATTTAGTGGTGGAGAGTAGAAGT  R: CCCTCCAAAACAAAAAATACTCTAATTATA  S: CCCCAACCCTCCAACC |
|  | Primer2 | F: GGAGGGGGTTGTTAATATTGATT  R: [Btn]AATCACTAATACCCAAAATTCTCTT  S: GGGTTGTTAATATTGATTTAGTT |
| IGF2-  DMR1 | Primer1 | F: GTTTGTATGTTTTGGTGGTTTTTTAATGG  R: [Btn]CTACTTTCTACCCTCCAAAAATCT  S: GGAAAGTTTAGGAGTTTAT |
| IGF2-  DMR2 | Primer1 | F: TGGTTTTTTTGGAGATATATTGTGT  R: [Btn]ATCTCTTTATCTCACCCCATAATT  S: TTTTTGGAGATATATTGTGTTAT |
|  | Primer2 | F: [Btn]TGGTTTTTTTGGAGATATATTGTGT  R: TACCCCAAATCAAAAAATAAATAATCTCTT  S: CCTTCAACCCCACCT |
| IGF2/H19 ICR | Primer1 | F: [Btn]AAGGGTTATGGGGTGGTAT  R: ACACAAATACCTAATCCCTTTATTAAACCT  S1: CATTTAACTATAACCAAATCTACAC  S2: ACCTAAAATACTCAAAACTT |

Btn: biotinylated primer; F: Forward Primer; R: Reverse Primer; S: Sequencing Primer

**Supplementary Table 3** **Nucleotide sequences of primers (mouse) used for Chip-qPCR**

| Gene | Primers (5’ to 3’ direction) |
| --- | --- |
| DNMT3A | F: AAGCTCCCCAAAACAGAACG |
|  | R: GAAAGCGTGTGTGTCTGTGT |
